# Prevalence of Stroke-Associated Pneumonia and Its Predictors Among Hyperglycaemia Patients During Acute Ischemic Stroke

**DOI:** 10.7759/cureus.52574

**Published:** 2024-01-19

**Authors:** Marwa Elhefnawy, Norsima Nazifah Sidek, Siti Maisharah Sheikh Ghadzi, Baharudin Ibrahim, Irene Looi, Zariah Abdul Aziz, Sabariah Noor Harun

**Affiliations:** 1 School of Pharmaceutical Sciences, Universiti Sains Malaysia, Penang, MYS; 2 Clinical Research Centre, Hospital Sultanah Nur Zahirah, Terengganu, MYS; 3 Faculty of Pharmacy, Universiti Malaya, Kuala Lumpur, MYS; 4 Clinical Research Centre, Hospital Seberang Jaya, Seberang Jaya, MYS

**Keywords:** hyperglycaemia, unfavourable functional outcomes, mild acute ischemic stroke (ais), stroke-associated pneumonia, functional outcome

## Abstract

Background: Hyperglycaemia (HG) during an acute ischemic stroke (AIS) is not only associated with unfavourable functional outcomes but also associated with stroke-associated pneumonia (SAP). This study aimed to determine the prevalence of SAP among Malaysian patients with AIS and the predictors of SAP among patients with HG during AIS.

Methods: This is a retrospective cross-sectional study that included patients with AIS admitted to Hospital Sultanah Nur Zahirah, Malaysia from 2017 to 2020. SAP was defined as infection with pneumonia during the first seven days after IS. HG was defined as a blood glucose level > 7.8 mmol/L within 72 h after admission. Patients with SAP were divided into two groups according to HG status. Multivariate logistic regression analysis was performed using SPSS software, version 22 (IBM Corp., Armonk, NY) to identify SAP predictors among patients with HG. Kaplan-Meier log-rank test was used to compare the survival rate from unfavourable functional outcomes between hyperglycaemic patients with and without SAP.

Results: Among 412 patients with AIS, 69 (16.74%) had SAP. The prevalence of SAP among patients with HG and normoglycemia during AIS was 20.98%, and 10.65%, respectively. Age above 60 years, leucocytosis, and National Institute of Health Stroke Scale (NIHSS) > 14 on admission were independent predictors of SAP with aOR of 2.08 (95% CI;1.01-4.30), 2.83 (95% CI; 1.41-5.67), and 3.67 (95% CI; 1.53-8.80), respectively. No significant difference in unfavourable functional outcomes survival was found among patients with and without SAP (*p* = 0.653).

Conclusion: This study demonstrated the prevalence of SAP was higher among patients with HG compared to normoglycemia during AIS. The patient being old, leucocytosis and severe stroke upon admission predict the occurrence of SAP among patients with HG during AIS.

## Introduction

Stroke-associated pneumonia (SAP) is a frequent and serious complication of stroke [1]. It is defined as pneumonia occurring during the first seven days after stroke onset [2]. Its incidence has been reported to be approximately 2.3-44% [3-6]. Previous studies reported SAP was associated with a higher risk of mortality, a longer length of hospitalization, and poorer functional outcomes in stroke survivors [7-9]. The data on SAP prevalence is very sparse. There are apparent variations in reported prevalence between observational studies and within registries, where the prevalence of SAP in observational studies varied from 5.3 to 37.9%, while in registries varied from 6.7 to 30% [1,10]. In Malaysia, the SAP prevalence is inconclusive [11]. Karthigayan et al. [11] focused on elderly patients more than 65 years old and not subdividing stroke type (ischemic or haemorrhagic stroke). This unavailability of data on the disease burden in the Malaysian population may hinder decision-makers from establishing proper management strategies [12]. Thus, the prevalence of SAP among AIS patients in Malaysia needs to be reported for better disease management strategies.

Hyperglycaemia (HG) during acute ischemic stroke (AIS), which is defined as random blood glucose > 7.8 mmol/L during the acute ischemic attack was not only associated with poor outcomes but also associated with SAP [13,14]. HG was known to reduce the bactericidal ability of leukocytes, increasing the likelihood of pulmonary infection [15,16]. A modified version of the SAP prediction tool, which incorporated fasting HG, was found to be more effective in predicting SAP occurrence [16]. Nevertheless, diabetes status in determining the effect of HG on SAP was inconclusive. HG during AIS was significantly associated with the risk of SAP in patients without diabetes [17]. However, mortality of non-stroke-associated pneumonia was significantly correlated with HG during hospitalization but not with a history of diabetes [18]. Moreover, diabetes status itself has been postulated to have two distinct pathophysiologic entities of HG in AIS patients [19]. Thus, it is worth determining the effect of the diabetes status of SAP patients with HG, specifically during AIS.

All aforementioned studies investigated HG as one variable among different predictors for SAP [17,20]. However, no study investigated the prevalence of SAP and its predictors according to HG status during AIS. This could be important as HG and SAP occur during the acute phase of stroke with a high incidence rate. Moreover, both HG and SAP are associated with unfavourable functional outcomes [7,21,22]. This helps to identify at-risk patients by allowing them to control modifiable risk factors. Furthermore, this may help to accelerate the diagnosis process among at-risk patients and the management process to avoid untreated conditions. This study aims to determine the prevalence of SAP among Malaysian patients with AIS and the predictors of SAP among patients with HG during AIS.

## Materials and methods

Study population and design

Data of patients aged >18 years from January 2017 to December 2020, with a history of AIS was extracted from Hospital Sultanah Nur Zahirah (HSNZ), Kuala Terengganu, Malaysia. Stroke was diagnosed according to the World Health Organization’s criteria, which defines a focal (or at times global) neurological impairment of sudden onset and lasting more than 24 hours (or leading to death) and of presumed vascular origin [23,24]. All diagnoses were confirmed using brain computed tomography or magnetic resonance imaging. The ischemic stroke (IS) subtypes were defined using the Trial of ORG 10172 in Acute Stroke Treatment (TOAST) criteria into one of five categories based on risk factors, as well as clinical and brain imaging features: large artery atherosclerosis, cardioembolism, small vessel occlusion (lacunar), undetermined aetiology stroke or other aetiology [25,26]. SAP was diagnosed based on SAP diagnostic criteria by Centres for Disease Control and Prevention [2]. The stroke severity was evaluated on admission using the National Institute of Health Stroke Scale (NIHSS), the most commonly used neurological deficit rating scale with a maximum score of 42 [27]. The modified Rankin Scale (mRS) was used to assess functional outcomes upon discharge and during follow-up visits. The scale comprises seven levels, from 0 to 6 [28], with a higher score indicating a greater disability. A score of 0-2 is generally considered a good outcome, assuming an individual has complete functional independence. A mRS of 6 is often used to denote a deceased individual. In this study, the mRS score was dichotomised into two groups: no unfavourable functional outcomes (0-2) and unfavourable functional outcomes (3-5) [28]. Diabetes mellitus (DM) was defined either through the patient’s self-report, physician diagnosis or based on the use of hypoglycaemic medications prior to the index ischemic stroke, during hospitalization secondary to stroke or at discharge. Deceased patients with SAP data missing during AIS were excluded from this study. The calculated minimum sample size, n was 362 (http://www.raosoft.com/samplesize.html).

Investigated variables

In this study, demographic data such as gender, ethnicity, and educational status, either formal (graduate schools and colleges) or informal education, were collected. NIHSS as the physical examination tool was also included. Concomitant diseases, including DM, hypertension (HTN), hyperlipidaemia (HPLD), ischemic heart disease (IHD), and hyperuricemia were also obtained. The concomitant disease was defined either by physician diagnosis, patients’ electronic records, or deduced from the medication history, and the medications prescribed during discharge. In addition, other laboratory clinical data such as complete blood count (CBC), leucocytosis (white blood cells of more than 11.000 cells per microliter), high-density lipoprotein cholesterol (HDL-C), low-density lipoprotein cholesterol (LDL-C), triglyceride (TG), and total cholesterol (TC) level were included. The random blood glucose levels obtained 72 h after admission were reported.

The data were stratified into populations with normoglycemia and HG during AIS, then subdivided into two subgroups according to the SAP status. AIS patients with prior stroke DM were also selected to study predictors of SAP among these patients.

Measurement of blood glucose

Blood glucose was measured within 72 h after admission. HG was defined as a glucose level > 7.8 mmol/l [29-31]. Based on blood glucose levels within 72 h after admission, patients were distinguished into two groups: (1) normoglycemia and (2) hyperglycaemia. For the HG group, the patients were further divided into three subgroups: (1) short persistent HG (SPHG), HG at the time of admission and at a random time within 24 h after admission; (2) long persistent HG (LPHG), HG at the time of admission and throughout the 72 h of admission and (3) delay HG, HG developed 24 h after admission. Patients who received insulin during the first 72 h of admission based on their medical records were also included in the study. They were divided into two subgroups according to the onset of insulin therapy: (1) early receiver of insulin subgroup, which included patients who received insulin during the first hours of HG onset, and (2) late receiver of insulin subgroup, which included those who received insulin after 24 h of HG onset.

Analysis

Statistical analyses were performed using SPSS version 22.0 (IBM Corp., Armonk, NY). The categorical variables were presented in percentages. A comparison between SAP and no-SAP groups was analysed using Chi-square and Fisher’s exact tests for categorical variables. The significant variables associated with SAP among patients with AIS obtained from the univariate analysis were then included in the multivariate logistic regression. The association between significant variables and SAP was reported as an odds ratio (OR) with a 95% confidence interval (CI). Kaplan Meier log-rank test was used to compare the unfavourable functional outcome after discharge among patients with HG during AIS with and without SAP. In order to minimize bias from missing data, the pattern of missing values of independent variables was analysed. Multiple imputations were used to handle variables with missing values above 5%. Missing values in HDL-C, LDL-C, and total cholesterol levels during the AIS event were imputed from multiple imputation methods. Five imputations were used and Rubin’s rules were implemented to combine the results. A p-value of < 0.05 was considered statistically significant in both univariate and multivariate analyses.

Ethical approval

The ethical approval for this study was obtained from the Medical Research and Ethics Committee (MREC), Ministry of Health, Malaysia (Research ID: NMRR-20-1232-55354 (IIR)).

## Results

The general characteristics of the enrolled subjects are presented in Table 1. A total of 412 patients with AIS have been extracted and included in this study with 69 (16.74%) developed SAP. Among 69 patients with SAP, 51 (73.91%) patients were reported to have HG during AIS, where 50 (72.46%) of them were aged above 60 years and 40 (57.97%) were males. The majority of patients in this group had concomitant hypertension and pre-stroke DM, 52 (75.36%) and 35 (50.72%) respectively. Among patients with SAP, only 19 (27.53%) and 15 (21.73%) had pre-stroke HPLD and IHD respectively. A high percentage of patients who developed SAP experienced leucocytosis, 34 (49.27%).

**Table 1:** Descriptive results of patients with SAP during AIS. Abbreviations: AF; atrial fibrillation, BUN; blood urea nitrogen, CKD; chronic kidney diseases, DM; diabetes mellitus, FHOS: family history of stroke, HG; hyperglycemia, HPLD; hyperlipidemia, IHD; ischemic heart disease, HDL-C; high-density lipoprotein cholesterol, HTN; hypertension, LDL-C; low-density lipoprotein cholesterol, mRS; modified Rankin Score, NIHSS; National Institute of Health Stroke Scale, SAP; stroke associated pneumonia. The data has been represented as N (%).

Variable	Patients with no-SAP during AIS n = 343 (83.25%)	Patients with SAP during AIS n = 69 (16.74%)
Gender (male)	197 (57.43%)	40 (57.97%)
Age > 60	142 (41.39%)	50 (72.46%)
Education		
Nil	61 (17.78%)	25 (36.23%)
Primary	113 (32.94%)	23 (33.33%)
Secondary	137 (39.94%)	13 (18.84%)
HTN	266 (77.55%)	52 (75.36%)
DM	165 (48.10%)	35 (50.72%)
No-DM	178 (51.89%)	34 (49.27%)
HG	192 (55.97%)	51 (73.91%)
HG pattern		
Early	45 (13.11%)	10 (14.49%)
Persistent	117 (34.11%)	30 (43.47%)
Late	30 (8.74%)	11 (15.94%)
HPLD	93 (27.11%)	19 (27.53%)
IHD	31 (9.03%)	15 (21.73%)
Recurrent ischemic stroke	69 (20.11%)	20 (28.98%)
Lacunar	251 (73.17%)	33 (47.82%)
NIHSS > 14 upon admission	39 (11.37%)	19 (27.53%)
Thrombolytic agent (alteplase)	9 (2.62%)	2 (2.89%)
High LDL-C	175 (51.02%)	24 (34.78%)
Hypertriglyceridemia	68 (19.82%)	7 (10.14%)
Hypercholesterolemia	160 (46.64%)	18 (26.08%)
Low HDL-C	268 (78.13%)	50 (72.46%)
High BUN	89 (25.94%)	26 (37.68%)
mRS > 2	176 (51.30%)	51 (73.90%)

In the group of patients who had no SAP during AIS, 343 patients, 197 (57.43%) were males, 142 (41.39%) were aged above 60 years and 113 (32.94%) received primary education. One-hundred and sixty-five (48.10%) of the patients with no SAP had DM prior to the stroke. Ninety-three (27.11%) and 31 (9.03%) of no SAP group had HPLD and pre-stroke IHD respectively.

The percentage of patients with an NIHSS score > 14 upon admission in both SAP and no-SAP groups was 19 (27.53%) and 39 (11.37%) respectively. A similar trend was reported in the mRS score during the follow-up visit after discharge (median 88 days after discharge), where 51 (73.9%) and 176 (51.3%) of patients showed mRS > 2 in SAP and no-SAP groups, respectively.

Factors associated with SAP among patients with HG during AIS

Being older age (> 60 years) [aOR: 2.08 (95% CI; 1.01-4.30)], developed leucocytosis [aOR: 2.83 (95% CI; 1.41-5.67)], and NIHSS > 14 [aOR: 3.67 (95% CI; 1.53-8.80)] during the AIS significantly increased the odds of SAP in HG group (Table 2).

**Table 2:** Factors associated with stroke-associated pneumonia (SAP) during AIS. Abbreviations: AIS; acute ischemic stroke, HG; hyperglycemia, IHD; ischemic heart diseases, LDL-C; low-density lipoprotein cholesterol, NIHSS; National Institute of Health Stroke Scale. The data has been represented as N (%) *, which indicates statistical significance at p < 0.05 in multivariate analysis.

Variable	No-SAP among patients with HG during AIS n = 192 (79.01%)	SAP among patients with HG during AIS n = 51 (20.98%)	p-value	Odd Ratio	p-value	No-SAP among patients with normoglycemia during AIS n = 151 (89.43%)	SAP among patients with normoglycemia during AIS n = 18 (10.65%)	p-value	Odd Ratio	p-value
Age > 60	95 (49.5%)	35 (68.6%)	0.015	2.08 (1.01-4.30)	0.047*	71 (47.0%)	15 (83.3%)	0.004	3.44 (0.90-13.17)	0.071
Lacunar	138 (71.9%)	27 (52.9%)	0.01	0.637 (0.31-1.28)	0.210	113 (74.8%)	6 (33.3%)	0.0002	0.17 (0.05-0.54)	0.003*
High LDL-C	98 (51.0%)	15 (29.4%)	0.006	0.77 (0.27-2.17)	0.628	-	-	-	-	-
Hypertriglyceridemia	55 (28.6%)	6 (11.8%)	0.013	0.37 (0.13-1.06)	0.064	-	-	-	-	-
Hypercholesterolemia	95 (49.5%)	12 (23.5%)	0.001	0.69 (0.22-2.11)	0.520	-	-	-	-	-
Leucocytosis	58 (30.2%)	27 (52.9%)	0.002	2.83 (1.41-5.67)	0.003*	-	-	-	-	-
NIHSS > 14 upon admission	19 (9.9%)	15 (29.4%)	0.0003	3.67 (1.53-8.80)	0.004*	-	-	-	-	-
IHD	-	-	-	-	-	9 (6.0%)	5 (27.8%)	0.002	6.23 (1.48-26.28)	0.013*

No significant difference was observed between early and delayed insulin initiation in association with SAP among AIS patients with HG (Table 4). In contrast, in the normoglycemic group, lacunar and pre-stroke IHD were retained as the significant variables in the final model. Lacunar IS significantly decreased the odds of SAP in the normoglycemic group with aOR of 0.17 (95% CI; 0.05-0.54) while pre-stroke IHD significantly increased the odds of SAP in this group with aOR of 6.23 (95% CI: 1.48-26.28) (Table 2). Age above 60 years old [aOR: 3.12 (95% CI; 3.12(1.28-7.59)] and NIHSS > 14 [aOR: 4.67(95% CI; 1.59-13.74)] were associated and significantly increased the odds of SAP among DM patients with HG during AIS (Table 3).

**Table 3:** : Factors associated with SAP during AIS among patients with prior stroke DM. Abbreviations: AIS; acute ischemic stroke, HG; hyperglycemia, HDL-C; high-density lipoprotein cholesterol, NIHSS; National Institute of Health Stroke Scale. The data has been represented as N (%) *, which indicates statistical significance at p < 0.05 in multivariate analysis.

Variables	No-SAP among DM patients with HG during AIS n = 149 (%)	SAP among DM patients with HG during AIS n = 33 (%)	p value	Odd Ratio	p value
Age > 60	68 (45.6%)	24 (72.7%)	0.005	3.12 (1.28-7.59)	0.012*
Hypertriglyceridemia	51 (34.2%)	5 (15.2%)	0.037	0.40 (0.13-1.18)	0.097
Low HDL-C	24 (16.1%)	11 (33.3%)	0.023	0.46 (0.18-1.14)	0.095
NIHSS > 14 upon admission	13 (8.7%)	8 (24.2%)	0.012	4.67 (1.59-13.74)	0.005*

Risk factors of unfavourable functional outcomes (mRS > 2) after discharge

**Figure 1:**
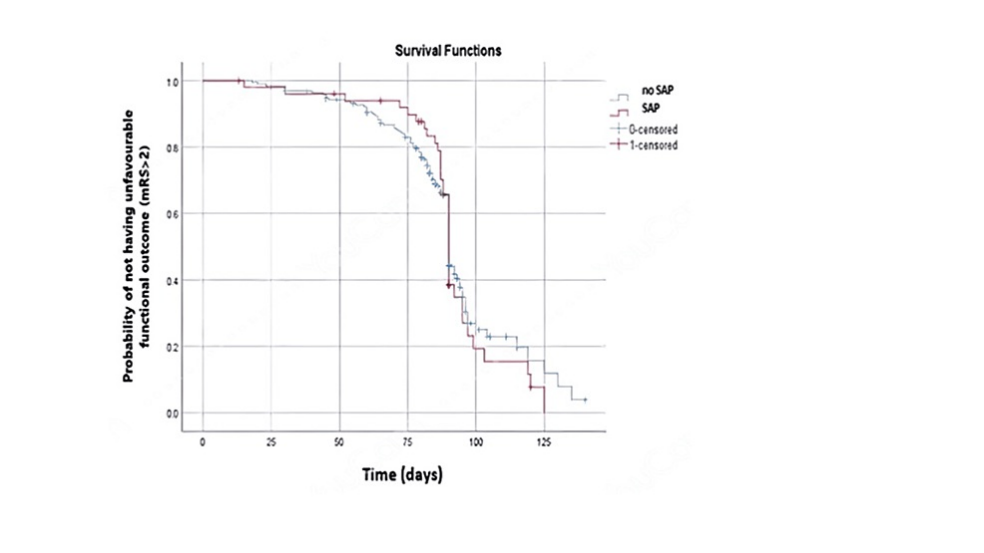
Probability of not having unfavourable functional outcome (mRS > 2) among patients with and without SAP Kaplan–Meier cumulative (cum) survival curve for freedom from unfavourable functional outcomes by log-rank test. SAP is shown in red and no-SAP is shown in blue. The follow-up interval is in days. (p = 0.635).

Based on the log-rank statistics analysis, there was no significant difference (p = 0.635) in survival rate according to the unfavourable functional outcome (mRS > 2) after discharge between SAP and no-SAP patients despite the SAP group showing an earlier (91.21 days after admission) and lower probability of not having an unfavourable functional outcome than the no-SAP group (92.47 days) (Figure 1).

## Discussion

To our knowledge, this is the first study to report the prevalence of SAP among patients with HG during AIS in the Malaysian population. SAP is one of the most problematic complications during AIS, associated with a high mortality rate [32]. Acute stroke gravitates the alteration of peripheral immune responses, resulting in transient lymphopenia and monocyte deactivation, increasing susceptibility to infections [33]. Stroke immunomodulation alters tracheal epithelium, reduces pulmonary clearance, and impairs secretion drainage, which plays a significant role in developing pneumonia [8]. In the current study, the incidence of SAP increased among IS patients with HG. This finding was consistent with a recent study that identified HG as an independent risk factor for SAP [20]. HG state reduces the bactericidal ability of leukocytes and, therefore, increases the likelihood of pulmonary infection [15,16]. Previous studies showed an augmentation of pneumonia is attributed to the presence of risk factors such as stroke severity, loss of consciousness, advanced age, and pre-existing comorbidities [8,32,33].

In the current study, an age of more than 60 was an independent predictor for SAP among patients with HG during AIS. This finding aligns with previous studies that reported the association between an older age and a higher risk of poststroke pneumonia [8,34,35]. This could be reasoned as older people tend to have more impaired swallowing function (dysphagia) [36] and comorbidities [37] than younger people. As dysphagia increases the risk of aspiration, the risk of SAP is elevated [38]. Additionally, severe stroke (NIHSS > 14) was found to be one of the SAP-independent predictors among patients who had HG during AIS. Previous studies showed similar results, i.e., patients with high NIHSS scores were more likely to develop pneumonia after stroke [39,40]. Furthermore, it was found that leucocytosis is an independent predictor of SAP in agreement with a previous study that included leukocyte count as a predictor of SAP prediction [35]. The immunologic changes after IS may explain this finding. Post-IS vascular occlusion leads to oxygen deprivation and thus stimulates an inflammatory immune response, which results in WBC elevation as a marker of inflammation [41]. Such alteration in the systemic inflammatory response leads to poststroke immunodeficiency [42], therefore increasing the susceptibility to infections such as SAP [41,43].

A seven-year nationwide prospective research of 512,869 adults demonstrated an increased death risk after infection in patients with DM [44]. Impaired immune function during HG may be related to the increased susceptibility to infection and mortality in DM patients [45]. A previous study revealed that infection is an independent risk factor of in-hospital mortality for AIS patients with prior-stroke DM [46]. In this study, results obtained from multivariate analysis of SAP predictors among DM patients were in agreement with the results of patients with HG during AIS as the majority of patients with HG during AIS had prior-stroke DM.

As compared to other subtypes of stroke, lacunar stroke was considered to be relatively benign due to a lower stroke severity at the initial presentation and early case fatality [47,48]. A previous study showed lacunar infarcts were associated with short-term good prognosis with decreased mortality and dependency [49]. Moreover, the non-lacunar stroke was previously recorded as an SAP-independent predictor [8]. In this study, SAP significantly decreased in patients with lacunar stroke in comparison to other stroke subtypes.

Surprisingly, the survival analysis reported no significant difference in survival rate based on the unfavourable functional outcome (mRS > 2) after discharge between SAP and no-SAP patients with HG during AIS. This result contradicts previous findings that reported a positive association between SAP and poor functional outcomes [7-9]. Thus, the influence of SAP on functional outcomes may be compromised by HG influence. Further studies are needed to verify this assumption.

Limitations

Since this is a retrospective study where the data was extracted from Hospital Sultanah Nur Zahirah (HSNZ), Kuala Terengganu, Malaysia, a single-centre study design remained one of the limitations of this study. Further external validation cohorts will be required to verify these results. As the first study to investigate the predictors of SAP among Malaysian patients with HG during AIS, this study may shed some light on the predictors of poor outcomes among patients with HG during AIS who developed SAP. However, no other pieces of information apart from mRS scores were collected on clinical conditions at discharge, thus limiting the possibility of excluding their influence on the measures of three-month follow-up. Further studies are needed to investigate different doses of insulin influence on SAP prevalence and outcomes among patients with HG during AIS.

## Conclusions

The prevalence of SAP among patients with HG is two times higher than those with normoglycemia. The predictors for SAP among patients with HG during AIS were age > 60, leucocytosis, and NIHSS > 14. These findings highlight the increased risk of SAP among patients with HG during acute ischemic stroke. Moreover, they may guide clinicians in establishing proper follow-up and management strategies based on the risk factors in patients.

## Appendices

**Table 4:** Univariate analysis of variables associated with SAP among DM patients with HG during AIS. Abbreviations: Abbreviations: AIS; acute ischemic stroke, BUN; blood urea nitrogen, CKD; chronic kidney disease, DM; diabetes mellitus, IHD; ischemic heart disease, IS; ischemic stroke, HDL-C; high-density lipoprotein cholesterol, HPLD; hyperlipidemia, HTN; hypertension, LDL-C; low-density lipoprotein cholesterol, LPHG; long persistent hyperglycemia, NIHSS; National Institute of Health Stroke Scale, SAP; stroke associated pneumonia, SPHG; short persistent hyperglycemia. The data has been represented as N (%). Significance at p < 0.05

Variable	No SAP among DM patients with HG during AIS n = 149 (%)	SAP among DM patients with HG during AIS n = 33 (%)	p value
Recurrent IS	12 (8.1%)	6 (18.2%)	0.079
HTN	137 (91.9%)	29 (87.9%)	0.455
IHD	21 (14.1%)	7 (21.2%)	0.296
HPLD	54 (36.2%)	8 (24.2%)	0.226
Gender male	78 (52.3%)	19 (57.6%)	0.700
Age >60	68 (45.6%)	24 (72.7%)	0.005
CKD	14 (9.4%)	4 (12.1%)	0.746
Lacunar	112 (79.4%)	19 (63.3%)	0.406
High LDL-C	74 (49.7%)	13 (39.4%)	0.285
Hypertriglyceridemia	51 (34.2%)	5 (15.2%)	0.037
Hypercholesterolemia	77 (51.7%)	11 (33.3%)	0.056
Low HDL-C	24 (16.1%)	11 (33.3%)	0.023
Leucocytosis on admission	47 (31.5%)	16 (48.5%)	0.065
Low haemoglobin	29 (19.5%)	9 (27.3%)	0.319
NIHSS > 14 upon admission	13 (8.7%)	8 (24.2%)	0.012
High BUN	48 (32.2%)	14 (42.4%)	0.351
Hyponatremia	12 (8.1%)	4 (12.1%)	0.855
Hypokalaemia	25 (16.8%)	9 (27.3%)	0.139
HG pattern SPHG LPHG Delay HG	27 (18.1%) 110 (73.8%) 12 (8.1%)	4 (12.1%) 26 (78.8%) 3 (9.1%)	0.461
Insulin during AIS	12 (8.1%)	4 (12.1%)	0.425

**Table 5:** Univariate analysis of variables associated with SAP among patients with HG and normoglycemia during AIS. Abbreviations: Abbreviations: AIS; acute ischemic stroke, BUN; blood urea nitrogen, CKD; chronic kidney disease, DM; diabetes mellitus, IHD; ischemic heart disease, IS; ischemic stroke, HDL-C; high-density lipoprotein cholesterol, HPLD; hyperlipidemia, HTN; hypertension, LDL-C; low-density lipoprotein cholesterol, LPHG; long persistent hyperglycemia, NIHSS; National Institute of Health Stroke Scale, SAP; stroke associated pneumonia, SPHG; short persistent hyperglycemia. The data has been represented as N (%). Significance at p < 0.05

Variable	No SAP among patients with HG during AIS n = 192 (%)	SAP among patients with HG during AIS n = 51 (%)	p value	No SAP among patients with normoglycemia during AIS n = 151 (%)	SAP among patients with normoglycemia during AIS SAP n = 18 (%)	p value
Recurrent IS	39 (20.3%)	16 (31.4%)	0.093	30 (19.9%)	4 (22.2%)	0.510
DM	149 (77.6%)	33 (64.7%)	0.059	16 (10.6%)	2 (11.1%)	0.599
HTN	166 (86.5%)	40 (78.4%)	0.156	100 (66.2%)	12 (66.7%)	0.970
IHD	22 (11.5%)	10 (19.6%)	0.126	9 (6.0%)	5 (27.8%)	0.002
HPLD	59 (30.7%)	14 (27.5%)	0.650	34 (22.5%)	5 (27.8%)	0.567
Gender male	105 (54.7%)	28 (54.9%)	0.978	92 (60.9%)	12 (66.7%)	0.636
Age >60	95 (49.5%)	35 (68.6%)	0.015	71 (47.0%)	15 (83.3%)	0.004
CKD	14 (7.3%)	5 (9.8%)	0.552	8 (5.3%)	2 (11.1%)	0.289
Lacunar	138 (71.9%)	27 (52.9%)	0.010	113 (74.8%)	6 (33.3%)	0.0002
AF	1 (0.5%)	2 (3.9%)	0.120	0 (0.0%)	1 (5.6%)	0.107
High LDL-C	98 (51.0%)	15 (29.4%)	0.006	77 (51.0%)	9 (50.0%)	0.567
Hypertriglyceridemia	55 (28.6%)	6 (11.8%)	0.013	13 (8.6%)	1 (5.6%)	0.546
Hypercholesterolemia	95 (49.5%)	12 (23.5%)	0.001	65 (43.0%)	6 (33.3%)	0.299
Low HDL-C	38 (19.8%)	15 (29.4%)	0.139	37 (24.5%)	37 (24.5%)	0.547
Leucocytosis on admission	58 (30.2%)	27 (52.9%)	0.002	26 (17.2%)	5 (27.8%)	0.331
Low haemoglobin	37 (19.3%)	14 (27.5%)	0.202	17 (11.3%)	2 (11.1%)	0.672
NIHSS > 14 upon admission	19 (9.9%)	15 (29.4%)	0.0003	20 (13.2%)	4 (22.2%)	0.238
High BUN	59 (30.7%)	22 (43.1%)	0.116	30 (19.9%)	13 (72.2%)	0.755
Hyponatremia	12 (6.3%)	5 (9.8%)	0.090	3 (2.0%)	1 (5.6%)	0.365
Hypokalaemia	32 (16.7%)	13 (25.5%)	0.352	26 (17.2%)	3 (16.7%)	0.950
HG pattern SPHG LPHG Delay HG	45 (23.4%) 117 (60.9%) 30 (15.6%)	10 (19.6%) 30 (58.8%) 11 (21.6%)	0.567			
Insulin during AIS Early received insulin Delay received insulin	84 (43.8%) 13 (6.8%)	19 (37.3%) 4 (7.8%)	0.657			
